# Overexpression of SRD5A3 in Hepatocellular Carcinoma and Its Molecular Mechanism: A Study of Bioinformatics Exploration Analysis with Experimental Verification

**DOI:** 10.1155/2022/7853168

**Published:** 2022-09-16

**Authors:** Erbao Chen, Jing Yi, Qingqi Ren, Yuanna Mi, Zhe Gan, Jikui Liu

**Affiliations:** ^1^Department of Hepatobiliary and Pancreatic Surgery, Peking University Shenzhen Hospital, Shenzhen 518036, Guangdong, China; ^2^School of Medicine, Southern University of Science and Technology, Shenzhen 518055, Guangdong, China

## Abstract

**Background:**

Hepatocellular carcinoma (HCC) is one of the most common cancers worldwide, and more prevalent among males than females. However, the biological role of enzyme 5*α*-reductase (SRD5A3), which plays a critical role in the androgen receptor signaling pathway during HCC development, remains poorly understood.

**Methods:**

ONCOMINE, GEPIA, UALCAN, and Kaplan–Meier Plotter were used to analyze the expression and prognostic value of SRD5A3 in HCC. STRING and Metascape were applied to analyze potential target and molecular pathways underlying SRD5A3 in HCC. A real-time quantitative reverse transcription-polymerase chain reaction was used to validate the downstream target expression of SRD5A3.

**Results:**

The expression of SRD5A3 was significantly overexpressed in HCC tissues compared with normal tissues, while the expression of SRD5A1 and SRD5A2 were downregulated in multiple public datasets. It may be that the low methylation of the SRD5A3 promoter leads to its overexpression. The level of SRD5A3 tended to be higher expressed in clinical samples with advanced stage and positive node metastasis. Furthermore, the patients with higher SRD5A3 were remarkably associated with poorer overall survival and disease-free survival in the TCGA data. In addition, the increased mRNA expression of SRD5A3 could predict poorer overall survival in Kaplan–Meier Plotter database including different patient cohorts. Moreover, HCC patients with higher level of SRD5A3 had significantly shorter recurrence-free survival, progression-free survival, and disease-specific survival. Furthermore, enrichment analysis demonstrated that multiple processes, such as steroid hormone biosynthesis, lipid biosynthetic process, and androgen metabolic process, were affected by SRD5A1-3 alterations. *In vitro* experiments showed that the expression of SRD5A3 was increased in HCC tissues than that in adjacent tissues. SRD5A3 silencing promoted the expression of DOLK in two HCC cell lines.

**Conclusions:**

This study identified SRD5A3/DOLK as a novel axis to regulate HCC development.

## 1. Introduction

Primary liver cancer is one of the major causes of cancer mortality globally, which ranks the second cause among males, and the sixth cause in females [1]. Hepatocellular carcinoma (HCC) represents the most common form of primary liver carcinoma. Chronic infection with hepatitis B or C virus (HBV or HCV) has been established to lead to the initiation and development of HCC [2, 3]. Tumor suppressor p53 is a well-known guardian of the genome to prevent oncogenic mutations. Overall, 50% of human cancer carried p53 mutation, p53 mutation promoted multiple cancer types cell growth, survival, and metastasis to maintain the malignant behavior of cancer cells. Although single base mutation in TP53 occurs in approximately 25% of HCC [4], many literature revealed that p52 mutation is one of the most causes of HCC tumorigenesis and progression [5, 6].

HCC is a malignant tumor with a high incidence in men. Females have significantly lower HCC incidence than males, with a women-to-men incidence ratio ranging from 4 : 1 to 7 : 1 [7]. Furthermore, males with HCC had a worse prognosis compared with female patients [8, 9]. The obvious sex disparity of HCC progression demonstrated that sex hormones and/or their receptors may exert critical functions for the initiation and development of HCC [10]. Moreover, female mice with estrogen receptor alpha silencing lost their resistance to HCC, while a reduced incidence of HCC was observed in male mice without the androgen receptor (AR). AR signals have been reported to modulate malignant transformation and development of HCC [11]. These data indicated that active androgen and androgen-mediated signaling pathways may contribute to HCC [12, 13]. However, the results of a clinical trial using antiandrogens were not satisfactory, with few beneficial effects on patients [14]. Therefore, exploring other potential targets in the AR pathway becomes urgent. 5*α*-reductase is one of the important enzymes in AR signaling and converts testosterone to the more powerful androgen dihydrotestosterone (DHT) [15]. Three isoenzymes of 5*α*-reductase have been identified and reported, which are the products of steroid 5*α*-reductase types I, II, and III encoded by srd5a1, srd5a2, and srd5a3, respectively [16]. SRD5A1 is expressed mainly in the skin, liver, and brain while SRD5A2 is a major enzyme in androgen targets organs such as the prostate and seminal vesicles [17]. Although SRD5A1 and SRD5A2 have some structural homology, they have markedly different kinetic parameters as well as chromosomal localizations [18]. Previous literature revealed that the expression of SRD5A1 and SRD5A2 is associated with the pathogenesis of polycystic ovary syndrome [19]. Dysregulated expression of SRD5A1 and SRD5A2 also were observed in prostate cancer [20]. SRD5A3 was originally identified in hormone-refractory prostate tissue by genome-wide gene expression profiles and SRD5A3 inhibition also suppressed the proliferation of prostate cancer cells [21]. Godoy et al. further found that overexpression of SRD5A3 protein in prostate cancer compared with benign tissues [22]. These observations demonstrated that SRD5A3 might play important roles during prostate cancer growth and progression. Except for the oncogene role in prostate cancer, recent researchers suggested that SRD5A3 may play an important role in protein *N*-linked glycosylation. SRD5A3 serves as a polyprenol reductase and promotes the reduction of polyprenol to dolichol [23]. Mutations of SRD5A3 were associated with congenital disorders [24] and Kahrizi syndrome [25]. Previous literature showed that SRD5A3 could promote tumor proliferation of HCC. So far, there is little systematical analysis of the expression profiles and prognostic value of the SRD5A1-3 family in patients with HCC.

The purpose of the current study was to systematically analyze the expression and clinical value of the SRD5A1-3 family in patients with HCC. The transcriptional level of SRD5A1-3 was investigated in both HCC tissues and normal tissues by public datasets. Relevant clinical information was analyzed between low and high-expression groups. Then, the significance of SRD5A1-3 in predicting prognosis for HCC was analyzed based on the GEPIA database and the Kaplan–Meier plotter database, and later the gene-gene interaction networks were constructed for SRD5A1-3 to explore the underlying mechanisms. Lastly, experiments *in vitro* were performed to explore the expression and potential target of SRD5A3 in clinical HCC tissues and cell lines. Therefore, the present study aimed to explore the expression and clinical value of SRD5A3, so as to provide a new approach for further specific treatment for HCC.

## 2. Materials and Methods

### 2.1. ONCOMINE Analyses

The online cancer microarray database and ONCOMINE (https://www.oncomine.org/) database [26] were used to evaluate the transcriptional levels of SRD5A1-3 genes among various cancer types. The mRNA expression of SRD5A1-3 was compared between tumor tissues and normal specimens. We entered the gene name “SRD5A1,” “SRD5A2,” and “SRD5A3” and obtained the all results generated by the ONCOMINE dataset. *P* value was calculated by the Student's *t*-test, and the significance of *P* value was set at 0.05.

### 2.2. The Gene Expression Profiling Interactive Analysis (GEPIA) Analysis

The online database Gene Expression Profiling Interactive Analysis (GEPIA) [27] is a designed interactive web database that contains 9736 tumors and 8587 normal specimens from the TCGA and the Genotype tissue expression (GTEx) projects, which are used to analyze mRNA sequencing expression. Notably, GEPIA allows to offer prognostic analyses (overall survival and disease-free survival) on HCC patients. The cutoff for higher or lower expression of SRD5A1-3 was set with the median expression of whole samples and survival plots were drawn out by GEPIA.

### 2.3. UALCAN Analysis

Online database UALCAN (https://ualcan.path.uab.edu) [28] provides easy access to the publicly available cancer TCGA database including 31 cancer types. It provides convenient scatter graphs and plots depicting the relationship between gene expression and clinical features. In this study, we used this database to investigate the mRNA expression of SRD5A1-3 in HCC tissues and their adjacent normal tissues as well as cancer stages, node metastasis status, and TP53 mutation status. *P* value with significance was set at 0.05.

### 2.4. Kaplan–Meier Plotter Database

Kaplan–Meier Plotter (https://www.Kmplot.com) [29] was used to evaluate the prognostic value of the mRNA expression level of SRD5A3 gene. For analyzing HCC patient overall survival, postprogression survival, progression-free survival, and disease-specific survival, all specimens were separated into two groups (high vs. low, cutoff: 50% median expression level). The hazard ratio (HR) with 95% confidence intervals and log-rank *P* value were generated by the database. We acquired the K-M survival curves based on the value of the SRD5A3 probe, and the number at risk was exhibited below the curves.

### 2.5. Bioinformatics Analyses and Functional Enrichment

Metascape (https://metasape.org) [30] is an extract-rich annotation online tool and its pathway enrichment analysis made use of Gene Ontology (GO), Kyoto Encyclopedia of Genes and Genomes (KEGG), Reactome, MSigDB, and so on. The functional enrichment analysis of SRD5A3 gene was carried out by Metascape based on molecular function, biological processes, and singling pathway. The protein-protein interaction networks were generated by the Retrieval of Interacting Genes (STRING) database (https://string-db.org/) to explore biological processes and specific pathways of SRD5A3.

### 2.6. Cell Lines, Cell Transfection, RNA Isolation, Real-Time Quantitative Reverse Transcription-Polymerase Chain Reaction (RT-PCR) and Clinical Samples

PLC/PRF/5 cell line was purchased from the Guangzhou Cellcook Biotech Co., Ltd. (Guangdong, China). HCCLM3 was gifted by Prof. Zhou Zhengjun, Fudan University, Zhongshan Hospital. Small interfering RNA and control of SRD5A3 were purchased from RiboBio (Guangzhou, China). The siRNA-1 sequence for SRD5A3 was: Forward, 5′-GCAGTACTGTTTTGGACTT-3′; The siRNA-2 sequence for SRD5A3 was: Forward, 5′-GGTGGCTAGTGGTGACAAA-3′. Total RNA from 32 for HCC tissues and 32 for paired adjacent normal tissues and HCC cell lines were extracted by Trizol. RT-PCR was performed as previously described [31]. The primers used are listed as follows:

  SRD5A1: Forward, 5′-CTACGGGCATCGGTGCTTA-3′; Reverse, 5′-AATCGCCATTGTACACGCCA-3′;
  SRD5A2: Forward, 5′-TGCCTTCCTTCGCGGTG-3′; Reverse, 5′-AGTACACAAATGTCCTGTGGAA-3′;
  SRD5A3: Forward, 5′-CCTGCTGACCCTACTGCTG-3′; Reverse, 5′-AAAGTGGGAAAAATATCTCTTGGG-3′;
  AKR1C2: Forward, 5′-CCAGTGTCTGTAAAGGAGGACA-3′; Reverse, 5′-AGGCTTTGAGGATTCTGCACT-3′;
  AKR1C4: Forward, 5′-TGAAAGAAGTGGCAAGCAATGG-3′; Reverse, 5′-CTGTTCCTCGGAACCTCTGG-3′;
  DOLK: Forward, 5′- AATACAAGTGGGACCGGCTG-3′; Reverse, 5′-CCACAATGCCAAAACGCTCA-3′;
  HSD17B3: Forward, 5′-CCACAGAGATCGAGCGGAC-3′; Reverse, 5′-GCGTTCAGGAAATGGCTTGG-3′;
  GAPDH: Forward, 5′-AGCCACATCGCTCAGACAC-3′; Reverse, 5′-GCCCAATACGACCAAATCC-3′;

### 2.7. Ethics Statement

Our research protocol was approved by the Ethics Committee of the Peking University Shenzhen Hospital. 32 HCC tissues and adjacent peritumor tissues for validation were obtained from our center. All patients had given permission for their samples to be used in research and the samples were administered by the Peking University Shenzhen Hospital.

### 2.8. Statistical Analysis

Statistical analyses on the RT-PCR were analyzed in the GraphPad prism 9.0.0. The associations between the different groups and clinical features were assessed using the *χ*^2^ test or Fisher's exact test. The data were analyzed with the student's *t*-test and *P* value <0.05 was considered statistically significant.

## 3. Results

### 3.1. SRD5A3 Was Overexpressed in HCC Patients

**Figure 1:**
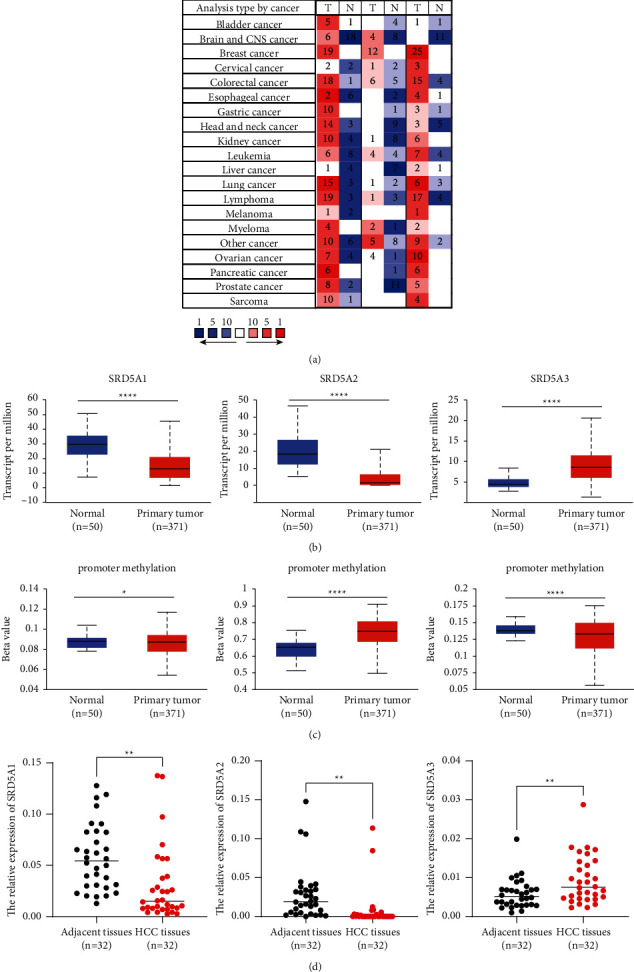
The overexpression of SRD5A3 in HCC tissues. (a) SRD5A expression at the transcriptional level among multiple cancer types based on the ONCOMINE database. (Color read means high expression level in a cancer sample, and color blue means low expression level in a cancer sample) (b) The expression profiles of SRD5A gene family in TCGA HCC cohort. (c) A significant correlation was found between the expression of SRD5A family genes and their promoter methylation. (d) The expression of SRD5A1-3 in HCC tissues from our center. ^*∗*^*P* < 0.05; ^*∗∗*^*P* < 0.01; ^*∗∗∗*^*P* < 0.001, ^*∗∗∗∗*^*P* < 0.0001.

Totally, three SRD5A genes (SRD5A1, SRD5A2, and SRD5A3) have been identified and reported in mammalian cells. In the present study, the ONCOMINE database was used to explore the expression of SRD5A gene transcriptional levels between cancers and normal tissues (Figure 1(a)). As shown in Figure 1, the expression of SRD5A3 was remarkably overexpressed in HCC tissues, while the level of SRD5A1 and SRD5A2 were significantly downregulated in HCC tissues [32–35]. The expression of SRD5A1 was decreased in tumors than that in normal tissues in the Mas liver, Roessler liver, and Roessler liver 2 datasets, while the level of SRD5A1 was increased in the Chen liver dataset. However, the above differences did not reach statistical significance. For SRD5A2, the results from four datasets showed the transcriptional level of SRD5A2 was downregulated in the tumor tissues. Wurmbach's dataset revealed a fold change of −9.467. Of note, the differences in SRD5A2 expression between HCC tissues and normal tissues were not reached statistical significance. Interestingly, the results from Wurmbach and Roessler datasets discovered the expression of SRD5A3 was upregulated with statistical significance in HCC tissues relative to normal tissues (Table 1). To validate our findings based on GEO datasets, we performed the analysis of SRD5A1-3 in the TGCA database. The expression of SRD5A1 was slightly downregulated in the cancer tissues relative to those within normal tissues. The level of SRD5A2 was significantly downregulated in the cancer tissues than that in normal tissues. The transcriptional level of SRD5A3 was significantly upregulated in the tumor tissues relative to normal tissues (Figure 1(b)). DNA methylation is a critical epigenetic modification mechanism and a potential target for cancer treatment. Until now, no literature has reported the correlation of SRD5A1-3 methylation with HCC. We found that SRD5A2 and SRD5A3 expression was negatively associated with promoter methylation of SRD5A2 and SRD5A3, respectively, while the expression of SRD5A1 was positively correlated with promoter methylation of SRD5A1 (Figure 1(c)). Those findings suggested that hypermethylation of SRD5A3 may inhibit the expression of SRD5A3 in promoting cancer development. To validate the bioinformatic results, we performed RT-PCR to explore the expression of SRD5A1-3 based on 32-pair clinical samples from our hospital. We observed that the expression of SRD5A3 was highly expressed in the HCC tissues than that in the adjacent tissues, while the level of SRD5A1 and SRD5A2 were downregulated in the HCC tissues (Figure 1(d)).

### 3.2. Correlation between SRD5A1-3 and Clinicopathological Parameters

**Figure 2:**
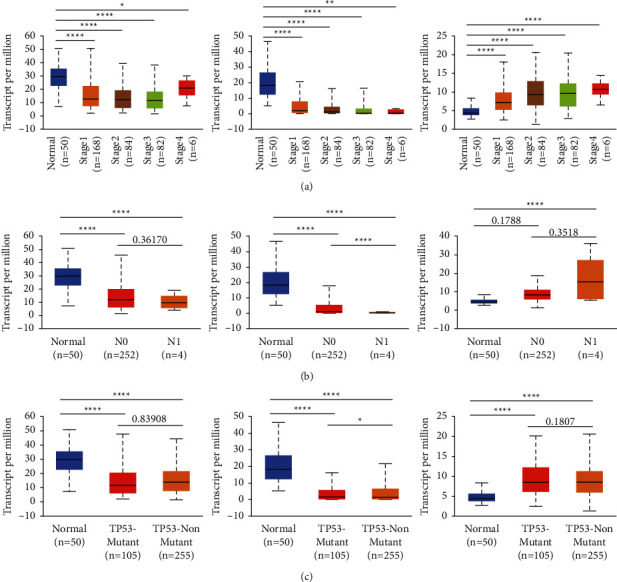
Correlation between mRNA expression of SRD5A1-3 in HCC analyzed using UALCAN. (a)-(b) The high level of SRD5A3 was associated with more advanced (a) tumor stage (stage 3 > stage 2 > stage 1) and (b) positive node metastasis (*N*2 > *N*1 > *N*0). (c) The expression of SRD5A1/2/3 was not associated with the TP53 mutation status. ^*∗*^*P* < 0.05; ^*∗∗*^*P* < 0.01; ^*∗∗∗*^*P* < 0.001, ^*∗∗∗∗*^*P* < 0.0001.

To illustrate the relationship between SRD5A1-3 and clinical features in HCC, the relationship between SRD5A1-3 expression and tumor stage was analyzed. The expression of SRD5A1and SRD5A2 was negatively correlated with the HCC tumor stage, and the high expression of SRD5A3 was significantly positively associated with the high tumor stage (Figure 2(a)). Interestingly, the bioinformatics analysis showed that the level of SRD5A3 tended to be upregulated in more advanced tumor stages (stage 4 > stage 3 > stage 2 > stage 1) and positive node metastasis (*N*2 > *N*1 > *N*0) (Figure 2(b)). Additionally, we found that the expression of SRD5A1/2/3 was not associated with the TP53 mutation status (Figure 2(c)).

### 3.3. Higher Level of SRD5A3 Was Associated with Several Different Clinical Outcomes in HCC

**Figure 3:**
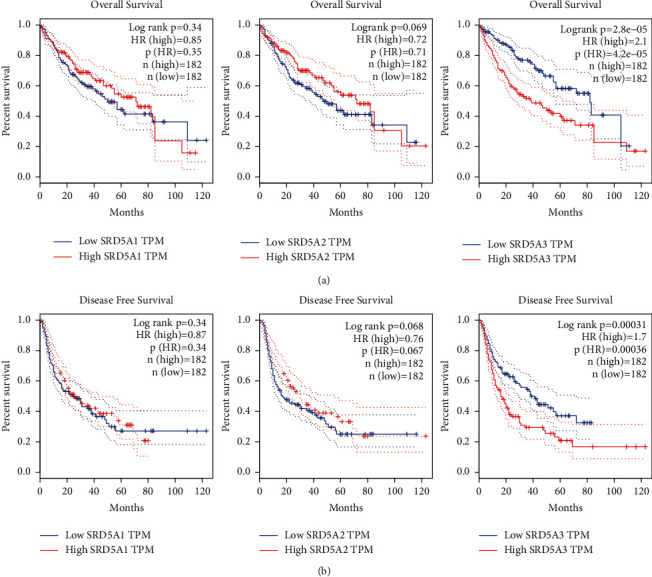
The impact of the SRD5A gene family on several clinical outcomes. (a) Significant SRD5A gene family transcriptional levels in predicting the overall survival for patients with HCC. (SRD5A1, *P*=0.34; SRD5A2, *P*=0.069; SRD5A3, *P*=0.000028) (b) Significant SRD5A gene family transcriptional levels in predicting the disease-free survival for patients with HCC (SRD5A1, *P*=0.34; SRD5A2, *P*=0.68; SRD5A3, *P*=0.00031).

**Figure 4:**
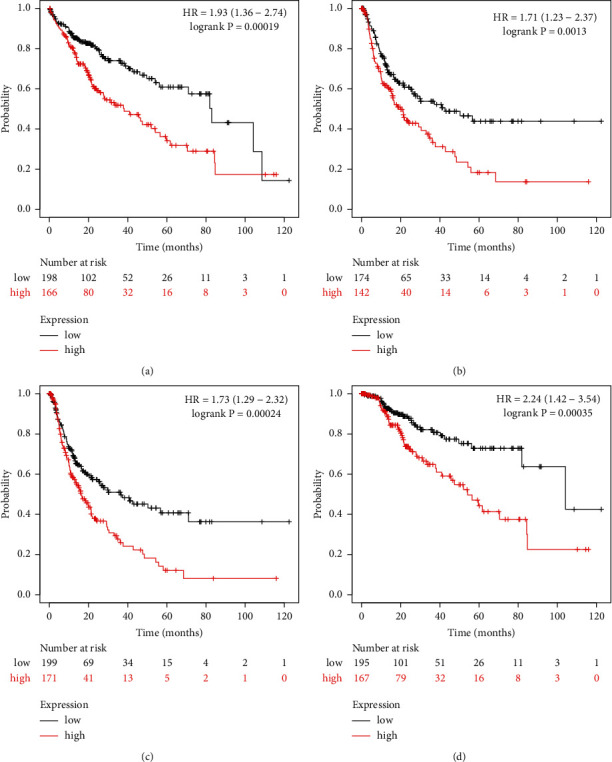
Survival curves showing the overall survival (OS) times of patients with HCC (Kaplan–Meier plotter). The elevated expression of SRD5A3 was positively associated with (a) OS, (b) DFS, (c) DSS, and (d) PFS.

Next, to assess the prognostic value of SRD5A mRNA expression in patients with HCC, survival analysis was performed. The group cutoff for high or low SRD5A1-3 expression was set with the median expression. As shown in Figures 3(a) and 3(b), only the expression of SRD5A3 was positively correlated with patients' overall survival and disease-free survival, while the expression of SRD5A1 and SRD5A2 did not were associated with the patient's clinical outcome. The above results suggested that among the SRD5A1-3 gene family, only the expression of SRD5A3 can predict HCC patients' survival, the Kaplan–Meier Plotter was further utilized to validate the prognostic value of SRD5A3. Consistent with previous findings, elevated expression of SRD5A3 was positively associated with OS and RFS (Figures 4(a) and 4(b)). Furthermore, patients with elevated expression of SRD5A3 had shorter disease-specific survival (DSS) and progression-free survival (PFS) (Figures 4(c) and 4(d)). In conclusion, SRD5A3 may act as a poor prognostic indicator for HCC.

### 3.4. Predicted Functions and Pathway Enrichment Analyses of SRD5A3 Genes Family

**Figure 5:**
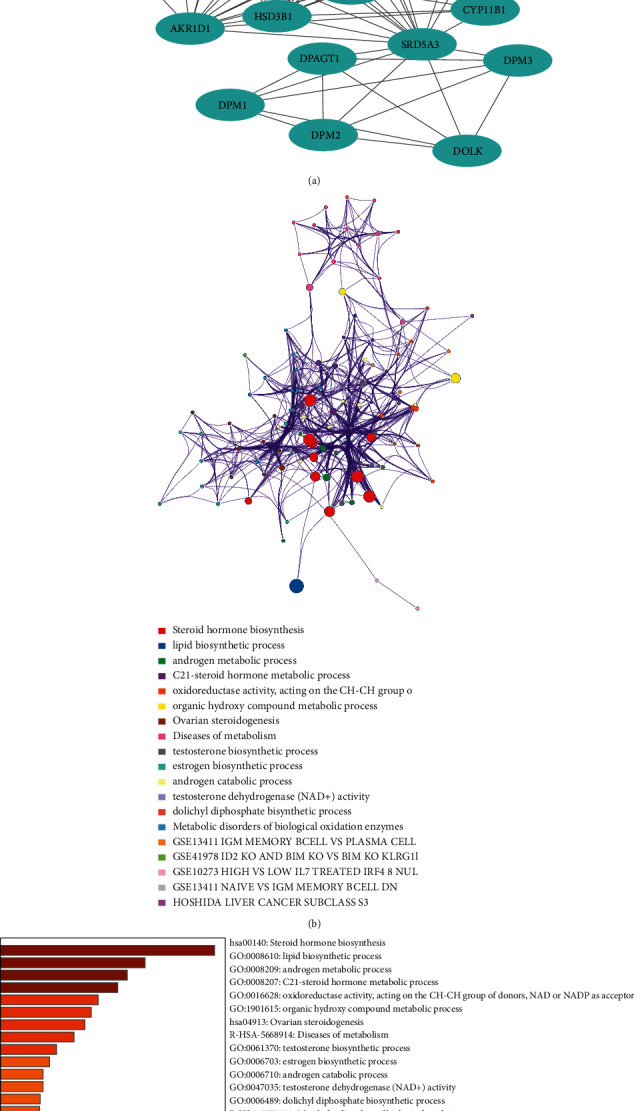
The protein-protein interaction network of SRD5A gene family. (a) SRD5A gene expression showed correlation with the expression of genes shown below: SRD5A3, SRD5A2, SRD5A1, HSD3B2, HSD3B1, HSD17B6, HSD17B3, HSD17B2, HSD17B1, DPM3, DPM2, DPM1, DPAGT1, DOLK, DHDH, CYP19A1, CYP17A1, CYP11B1, CYP11A1, AKR1D1, AKR1C3, AKR1C2, and AKR1C1. (b) The network of enriched GO terms. Nodes represent GO terms, and node size indicates the number of genes involved. Nodes that share the same cluster are usually close to each other, and the thicker the edge, the higher the similarity. (c) GO enrichment analysis predicted three main functions, including biological process, cellular components, and molecular functions (*P* < 0.05), and each GO term is colored based on the value of −log10 (*P*-value).

Genes showing co-expression with the SRD5A1-3 gene family were examined using the STRING database. According to the results, all SRD5A1-3 gene expressions showed a correlation with the expression of genes shown below: SRD5A3, SRD5A2, SRD5A1, HSD3B2, HSD3B1, HSD17B6, HSD17B3, HSD17B2, HSD17B1, DPM3, DPM2, DPM1, DPAGT1, DOLK, DHDH, CYP19A1, CYP17A1, CYP11B1, CYP11A1, AKR1D1, AKR1C3, AKR1C2, and AKR1C1 (Figure 5(a)). Then, the lists of all the SRD5A1-3 genes expressed, together with linked genes displaying the highest alteration frequency, were compiled before they were analyzed by the GO approaches in Metascape (Figure 5(b)). Bioinformatics analysis suggested that the processes below were subjected to the influence of SRD5A gene alterations: steroid hormone biosynthesis, lipid biosynthetic process, and androgen metabolic process (Figure 5(c)).

### 3.5. Experimental Validation of SRD5A3 in HCC Cell Lines

**Figure 6:**
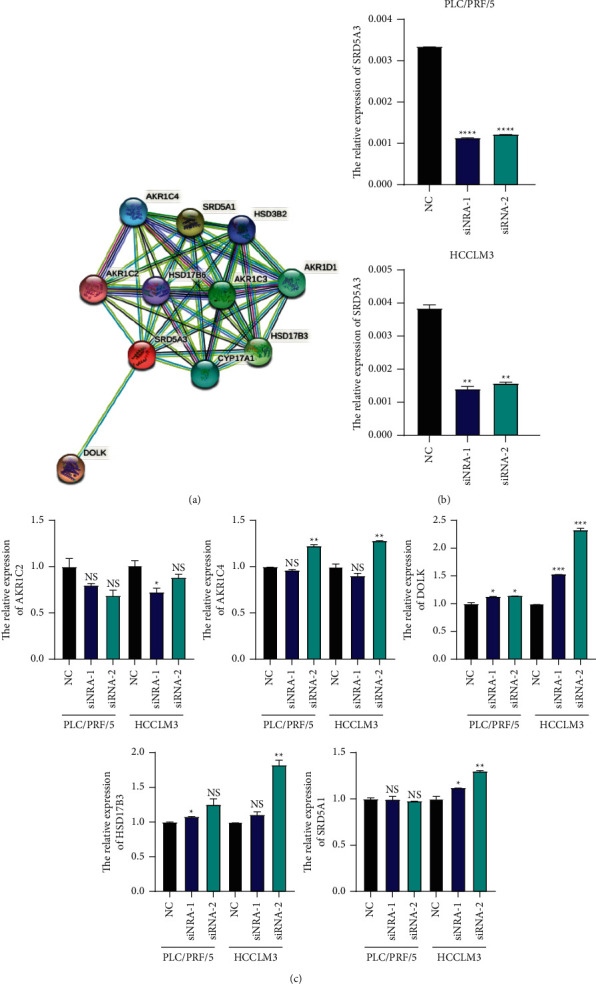
The experimental validation of SRD5A3 in HCC cell lines. (a) SRD5A gene expression showed a correlation with the expression of genes shown below: SRD5A1, HSD17B6, HSD17B3, HSD17B2, DOLK, CYP17A1, AKR1D1, AKR1C3, AKR1C2, and AKR1C4. (b) The knockdown efficiency of SRD5A3 through two siRNA was confirmed by RT-PCR. (c) RT-PCR revealed that the level of DOLK was consistently significantly increased by the SRD5A3 loss among five genes. ^*∗*^*P* < 0.05; ^*∗∗*^*P* < 0.01; ^*∗∗∗*^*P* < 0.001, ^*∗∗∗∗*^*P* < 0.0001, NS, no significance.

Among these potential targets, there were 10 targets of SRD5A3 in the STRING database (Figure 6(a)). We used two siRNA targeting SRD5A3 to inhibit its expression in PLC/PRF/5 and HCCLM3 cell lines (Figure 6(b)). Next, we performed RT-PCR to investigate the expression of random five genes (AKR1C2, AKR1C4, DOLK, HSD17B3, and SRD5A1). The results showed that only the level of DOLK showed consistent changes with statistical significance in the two HCC cell lines (Figure 6(c)). The above results indicated that DOLK was a potential target of SRD5A3 in HCC.

## 4. Discussion

In this study, the results of this study focused on the activity of testosterone-DHT transform and exhibited that the expression of SRD5A3 was overexpressed in the HCC tumor tissues. Notably, the high level of SRD5A3 among the SRD5A family can predict the survival of HCC patients. Furthermore, SRD5A3 silencing enhanced the expression of DOLK. Our observations have demonstrated that SRD5A3/DOLK axis is critical for HCC development.

Sex hormones, including androgens and estrogens and their corresponding receptors, as well as inflammatory responses and sex chromosomes, were involved in gender disparity in HCC [36]. Recent literature have been discovered that male hormones were increased in HCC. Androgens mainly served a critical role in various physiological and pathological processes by combing with AR. Abnormal expression of discrete sets of genes is frequently implicated in HCC gender disparity. However, few researchers investigated the role of different forms of androgens during HCC development. Indeed, the main finding of our study is that SRD5A1 and SRD5A2 decreased and SRD5A3 significantly increased in HCC tumor tissues. This study was therefore initiated to investigate whether SRD5A3 played an important part in HCC. For that, we further explored the clinical relevance of SRD5A3. The expression of SRD5A3 tended to be increasingly upregulated in the advance cancer stage. Previous literature revealed that SRD5A3 silencing inhibited HCC proliferation [37]. In this study, there are two main differences from previous studies. First, we not only analyze the value of SRD5A3 in HCC progression but also analyzed the value of SRD5A1 and SRD5A2 in HCC development. Secondly, we found that DOLK were new downstream of SRD5A3. Further experiments validated that the expression of DOLK was increased after SRD5A3 loss. These novel downstream targets would enrich our knowledge of the cancer-promoting mechanism of SRD5A3. The gene expression is closely negatively associated with promoter methylation. DNA methylation is an important mechanism for tumorigenesis. We discovered that the expression of the SRD5A gene family was negatively correlated with their promoter methylation. It may be that the low methylation of the SRD5A3 promoter leads to its overexpression. TP53 is the most frequently altered gene in multiple cancers. TP53 is a well-known tumor suppressor and works as a multifunctional transcriptional factor that controls cell fate. There was no significant correlation between SRD5A3 expression and TP53 mutation, indicating that the level of SRD5A3 was independent of the status of TP53 mutation.

To explore the potential targets of SRD5A3 in HCC progression, we observed that 10 genes interacted with SRD5A3 in the STRING dataset. The knockdown of SRD5A3 was constructed by two siRNA in the HCC cell lines. Moreover, we found that SRD5A3 loss promoted the level of DOLK in the two HCC cell lines. DOLK is involved in the synthesis of Dol-P-formation, and is an essential glycosyl carrier lipid for N- and O-linked glycosylation of proteins in the endoplasmic reticulum. However, the role of DOLK in cancer remains unclear.

An obvious limitation of this study is that the biological function of AR signals in the HCC initiation still remains controversial [38], and the conclusion from different studies suggested that AR might motivate tumorigenesis and cancer development at the early stage [39], yet might inhibit the tumor invasiveness at the advanced stages of HCC [40]. Whether inhibition of the AR pathway makes HCC patients benefit needs more experimental observation in the further.

In summary, our comprehensive bioinformation integrated approach identified that the transcriptional level of SRD5A3 was markable upregulated in HCC. This result was detected in clinical samples originating from GEO and TCGA populations which were analyzed by microarray hybridization and sequencing. In the mechanism, low methylation in the SRD5A3 promoter may lead to its overexpression. Moreover, overexpression of SRD5A3 can predict multiple types of poor survival. Experimental revealed that SRD5A3 knockdown enhanced the expression of DOLK in the two HCC cell lines. Therefore, SRD5A3/DOLK was a novel axis to regulate HCC development and presented a therapeutic target for HCC patients.

## Figures and Tables

**Table 1:** Remarkable SRD5A expression changes at transcription level between HCC cancer tissues and noncarcinoma counterparts (ONCOMINE database).

Genes	*T* vs. *N*	Fold change	*P* value	*t*-test	Reference or source
SRD5A1	104 vs. 76	1.184	0.122	1.167	Chen liver [32]
	38 vs. 19	−1.012	0.529	−0.073	Mas liver [33]
	225 vs. 220	−2.460	1.000	−13.891	Roessler liver2 [34]
	22 vs. 21	−2.891	1.000	−6.954	Roessler liver [34]

SRD5A2	35 vs. 10	−9.467	1.000	−15.344	Wurmbach liver [35]
	38 vs. 19	−1.648	0.999	−3.513	Mas liver [33]
	225 vs. 220	−6.034	1.000	−28.445	Roessler liver2 [34]
	22 vs. 21	−7.017	1.000	−10.387	Roessler liver [34]

SRD5A3	104 vs. 76	1.085	0.195	0.862	Chen liver [32]
	38 vs. 19	−1.001	0.509	−0.023	Mas liver [33]
	22 vs. 21	1.008	0.393	0.273	Roessler liver [34]
	35 vs. 10	1.446	**0.011**	2.528	Wurmbach liver [35]
	225 vs. 220	1.036	**0.032**	1.862	Roessler liver2 [34]

## Data Availability

The data used to support the findings of this study are available from the corresponding authors upon request.

## Abbreviations

HCC: Hepatocellular carcinoma
TCGA: The cancer genome atlas
GO: Gene ontology
KEGG: Kyoto encyclopedia of genes and genomes
TNM: Tumor size/lymph nodes/distance metastasis
GEO: Gene expression omnibus
GEPIA: Gene expression profiling interactive analysis
AR: Androgen receptor
HBV: Hepatitis B virus
HCV: Hepatitis C virus
DHT: Dihydrotestosterone
ARE: Androgen response elements
SRDA5: Steroid 5*α*-reductase
RT-PCR: Real-time quantitative reverse transcription-polymerase chain reaction
GTEx: Genotype tissue expression
DSS: Disease-specific survival
PFS: Progression-free survival
DOLK: Dolichol kinase.
